# A comparison of the nutritional content and price between dairy and non-dairy milks and cheeses in UK supermarkets: A cross sectional analysis

**DOI:** 10.1177/02601060221105744

**Published:** 2022-06-12

**Authors:** Alex Glover, Helen E. Hayes, He Ni, Vassilios Raikos

**Affiliations:** 1 Rowett Institute, 15553 University of Aberdeen, Aberdeen, Scotland, UK; 2 Guangdong Provincial Key Lab of Biotechnology for Plant Development, School of Life Sciences, 12451 South China Normal University, Guangzhou, China

**Keywords:** Dairy, milk, cheese, dairy alternatives, nutrient content, price

## Abstract

**Background:** Non-Dairy (ND) food consumption is rapidly increasing in the UK and for many consumers plant-based diets are presumed to be healthier than standard diets. ND alternatives have different nutritional compositions, and their consumption could present challenges on a public-health level. **Aim:** To compare the price and nutritional composition of dairy and ND milks and cheeses in UK supermarkets. **Methods:** Macro and micronutrient data was recorded from Alpro's website and the 6 leading UK grocers for their own-label ND milks and cheeses. For missing micronutrient values the McCance & Widdowson's dataset was used. 99 total products were extracted: 57 ND milks, 7 dairy milks, 10 dairy cheeses and 25 ND cheeses. Dairy milk and cheese were used as control against which all ND products were compared. **Results:** Soya and coconut milks had lower values of carbohydrates, sugars, calcium, iodine, and potassium (*p* < 0.01) than dairy. Almond milk had lower values of carbohydrates (*p* = 0.01), sugars, calcium, iodine, and potassium (*p* < 0.01) compared to dairy milk. Protein was significantly (*p* < 0.01) lower for all ND except soya. Dairy cheeses had higher values for energy, protein, iodine, potassium, riboflavin, vitamin B12 and calcium (*p* < 0.01) than ND. Median prices were similar between dairy and ND milks, whereas ND cheeses were significantly more expensive compared to dairy (*p* < 0.01). **Conclusions:** ND alternatives fall short in several key nutrients compared to dairy. Fortification, accurate labelling and nutrition education are needed to help consumers make healthy and informed choices.

## Introduction

Bovine milk and cheese are two of the most widely consumed foods worldwide and are both rich in a variety of essential nutrients. Dairy milk is the main source of iodine and calcium in the UK, and is also rich in potassium, riboflavin, and vitamin B12 (Watson et al., 2017). Dairy is also a source of high-quality protein and a nutrient dense food particularly important for children's cognitive development and bone growth (Garcia et al., 2019).

The recent demand for non-dairy (ND) milk and cheese alternatives in the UK has seen consistently high compounding annual growth rate, with cheese sales up 165% since 2018 and milk sales up 165% (Smart Protein Project, 2021). More people are consuming dairy alternatives largely due to environmental and health concerns of animal-based foods, with the United Nations urging a reduced level of consumption of dairy to protect the planet (Wickramasinghe et al., 2021). In 64% of occasions health is the key driver in consumption of ND products, which compared to 37% for dairy shows consumer awareness about the potential health benefits of ND (Kantar World Panel, 2016).

Epidemiological evidence suggests plant-based diets are associated with favourable health outcomes compared to standard omnivorous diets, with observed effects showing a reduction in markers of metabolic and cardiovascular diseases (CVD’s) (Hemler and Hu, 2019; Kaiser et al., 2021; Turner-McGrievy and Harris, 2014). These effects are associational and based on the consumption of whole plant foods. Few papers have examined these healthy plant-based diets with processed ND alternatives, which poses the question around their nutritional adequacy against dairy. Some literature has in fact challenged the completeness of ND milks compared to dairy (Chalupa-Krebzdak et al., 2018; Silva et al., 2020). Singhal et al. analyzed dairy and a selection of ND beverages in Australia, but only examined protein, calcium, and vitamin D (Singhal et al., 2017). Ma et al. found significantly lower iodine content in ND milks compared to dairy in the US market (Ma et al., 2016).

In the UK, micronutrient deficiency has been documented for a variety of population groups, including pregnant women (Bath et al., 2014; Knight et al., 2016). The 2018 national diet nutrition survey reported that 25% of women were below the lower reference nutrient intake for potassium and 10% for iodine (Derbyshire, 2018). With no iodine fortification programme currently planned in the UK, the decline in dairy consumption due to the replacement with alternative ND products, may represent a potential public health concern (Woodside and Mullan, 2020).

Existing research has not systematically examined the nutritional content of non-dairy milk and cheese alternatives available in UK supermarkets, including a wide range of micronutrients (Singhal et al., 2017). Thus, the aim of this study was to examine the macronutrient and micronutrient content of a selection of ND milks and ND cheddar alternatives from UK supermarkets using dairy counterparts as a control. In addition, price comparison between dairy and ND alternatives was included to provide a more comprehensive insight of the ND products available in the UK market.

## Methods

### Selection of products

For dairy milks, products included skim, semi-skimmed and whole milk variants. ND milks were categorized based on their botanical origin. ND milks were required to be free-from animal derived ingredients and included sweetened, unsweetened, fortified, unfortified and barista variants of almond, soya, oat, and coconut as these represent the majority of ND milks consumed in the UK. Any products formulated for life stages such as children's soya milk were excluded. Long life products were not included where a chilled version was available and were only included if they were the only product available. This was due to duplication, as all long-life products examined across retailer websites were identical to their chilled counterpart.

ND cheeses selected were all alternatives to cheddar, as this is the most widely consumed cheese in the UK as of 2021 (Statista, 2021). ND products were required to be free-from all dairy ingredients and described as a ‘cheddar alternative’ or clearly marketed as such. Inclusion criteria for cheeses were cheddar variants only including reduced fat, spiced, mature, sliced, grated and smoked variants. Specific food names from the database were ‘Cheese, Cheddar, English’, ‘Cheese Cheddar type, 30% less fat’ and ‘Cheese, Cheddar type, half fat’ to match grocer selection. This dataset is the industry standard and included OL supermarket products.

### Data collection from retailers

For milks, macro and micronutrient content including, energy, fat, saturates, carbohydrate, sugars, fibre, protein, salt, riboflavin, vitamin B12, iodine, potassium and calcium were extracted from market leader Alpro's website and all own-label ND & milk products from retailer's websites in the UK (Tesco, Sainsbury's, Asda, Morrison's, Aldi, and Lidl) between 3/05/21 to 18/05/21. Alpro had the largest proportion of non-dairy milks (28%) followed by Tesco (14%), Asda (12%), Morrison's (11%), Sainsburys (7%) and Oatley (9%). This represents almost 82% of the grocery market, correct as of April 2021. Tesco was used as a representative sample for dairy milks across grocers due to no observed differences in macronutrient content across grocers, and all using British milk for OL products.

For cheeses, brands included Violife, Applewood and Mexicana, and all the major four retailers (Tesco, Sainsbury's, Asda, Morrison's) own-label (OL) offerings, this represented >92% of the UK ND cheese market. Violife had the largest proportion of non-dairy cheeses (32%) followed by Asda & Morrison's (16%), Tesco, Sainsbury's & Mexicana (8%) and Mexicana (4%). Dairy cheeses were selected from UK market leader Tesco's OL range as a representative sample as all grocers used British milk as a base for their cheeses, and to avoid duplication in data collection.

Median price was calculated per L for milk and per 100 g for cheese for branded products across the retailers where it was available to account for differences in recommended selling price among retailers and promotional activity. Information from discount retailers Aldi and Lidl had to be extracted physically due to incomplete website content.

All nutritional values were cross-referenced with physical product labels for consistency. Micronutrient values were not available for soy, almond, coconut and oat ND products. Data was extracted from the McCance & Widdowson's dataset, and the values were calculated by taking into account the percentage of the botanical ingredient given on the label of each product. Similarly, potassium, iodine, riboflavin and vitamin B12 values were calculated for ND cheeses based on the % of coconut oil provided in the ingredients list. Modified starch in the ND cheese products makes up <25% and is such a highly processed excipient (typically just a filling agent) that its micronutrient content is considered negligible. The other ingredients constituted of salt, flavourings and colours, all not providing micronutrients.

### Statistical analysis

Analyzes were conducted per 100 mL or 100 g median averages data for milks and cheeses, with price, macronutrient and micronutrient content being compared using non-parametric tests due to data not being normally distributed, validated with Shapiro & Wilks. Non-dairy products were compared to dairy to test for statistical significance of at least *p* = 0.05. The Mann-Whitney test was used to compare cheese and non-dairy cheese, while the Kruskal-Wallis test was used to compare dairy and non-dairy milks. Statistical analysis was conducted in version 27 of IBM SPSS. Null hypothesis (H0)- there is no difference in the distribution of nutritional values for non-dairy products and dairy. Alternative hypothesis (H1)- the distribution of nutritional values for non-dairy and dairy is significantly different.

## Results

Sixty-four (64) dairy and ND products were extracted after eligibility screening and the following were included: 7 cow milks, 12 almond milks, 22 soya milks, 14 oat milks and 9 coconut milks. All but two soya products were chilled as these were the only variants of the product. Cheeses included 10 dairy cheeses and 25 ND cheeses.

### Nutrient composition and price- milks

**Figure 1.**
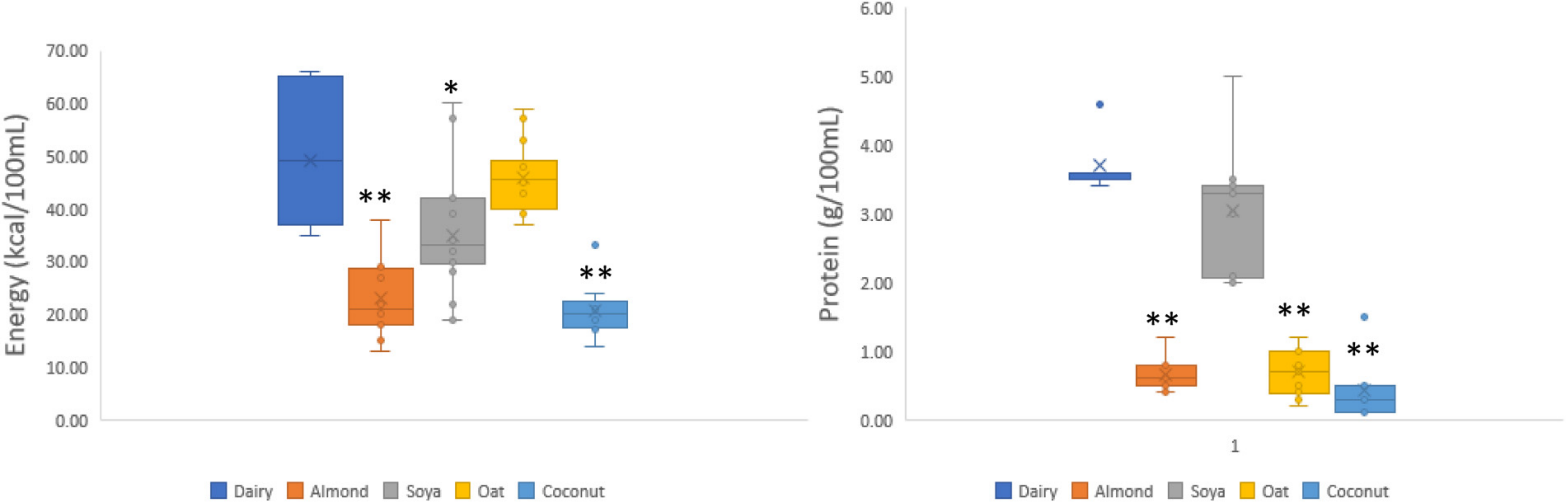
Energy and protein content of UK non-dairy milk alternatives. Data were analyzed using Kruskal–Wallis multiple comparison tests and are expressed as medians outlined with an X. Groups labelled with an asterisk (*) or two (**) are significantly different (*p* < 0.05 or *p* < 0.01 respectively) from the control median (dairy milk).

The composition of selected macro- and micronutrients present in dairy and ND milks, expressed as median and range per 100 mL, is presented in Table 1. Median values and ranges of energy and protein are presented in Figure 1, to highlight distributions of energy for dairy milk and protein for soya products. The price of ND milk didn't differ significantly to any of the dairy products (*p* = 0.125). Nevertheless, oat milk is available at higher cost (>60 pence) compared to all other ND alternatives and dairy. However, this effect was not statistically significant (*p* > 0.05). Total fat was not significantly different between ND milk products and dairy. Overall, ND milk substitutes contained lower levels of carbohydrates (except oat milk) and sugars and higher levels of fibre compared to dairy milk. Median values of energy, carbohydrates, sugar, and protein for almond and coconut were all significantly lower than dairy (*p* < 0.01). The median energy value for soya was significantly lower to dairy (*p* = 0.029) but protein levels were not significantly different (*p* = 1), unlike all other plant milks which were highly significantly lower compared to dairy (*p* < 0.01). On a micronutrient level, all ND milks were highly significantly lower to dairy regarding calcium, iodine, potassium, and vitamin B12 (*p* < 0.01). Riboflavin levels were not significantly different across groups (*p* = 1).

**Table 1.** Median and range of selected macro- and micronutrient content (per 100 mL) and price (per L) of dairy and ND milk alternatives in UK supermarkets.

Per 100 mL serving	Dairy	Range	Almond	Range	Soya	Range	Oat	Range	Coconut	Range
Fat (g)	1.7	1.7–3.7	1.5	1.5–3.1	1.8	1.1–4.3	1.5	0.5–3	1	0.5–1.4
Saturated Fat (g)	1	1–2.4	0.1	0.1–0.8	0.3	0.2–0.6	0.2 *	0.1–0.3	0.9	0.1–1.1
Carbohydrate (g)	4.8	4.8–5	1.2 *	1.2–3.2	1 **	0–3.1	6.6	5.5–9.7	2.2 **	0–5.3
Sugars (g)	4.8	4.8–5	0.5 **	0.5–3.2	0.5 **	0–2.7	4	0–6.1	1.9 **	0–3.3
Fibre (g)	0	0–0	0.5 **	0.5–0.8	0.5 **	0–1.6	0.8 **	0–1.4	0.1	0–0.5
Salt (g)	0.1	0.1–0.1	0.2	0.2–0.3	0.1	0–0.2	0.1	0–0.1	0.1	0.1–0.2
Calcium (mg)	124	124–124	120 **	120–121	120 **	7–123	120 **	5–124	120 **	120–122
Iodine (ug)	32	31–32	0 **	0–24.8	0 **	0–25	0 **	0–25	0 **	0–24.7
Potassium (mg)	162	161–162	15 **	15–15	43 **	43–43	37 **	37–222	14 **	14–14
Riboflavin (mg)	0.2	0.2–0.3	0.2	0.2–0.2	0.2	0–0.2	0.2	0–0.2	0 **	0–0.2
Vitamin B12 (ug)	0.9	0.9–0.9	0.4 **	0.4–0.4	0.4 **	0–0.4	0.4 **	0–0.4	0.4 **	0.4–0.4
Price (GBP) per L	0.9	0.7–1.2	0.9	0.8–1.8	0.9	0.6–2.1	1.6	0.8–2.0	1.0	0.8–1.8

One (*) and two (**) asterisks indicate statistically different values from the dairy milk control (*p* < 0.05) and (*p* < 0.01) respectively, as analyzed by Kruskal-Wallis tests. GBP (Great British pounds).

### Nutrient composition and price- cheeses

**Figure 2.**
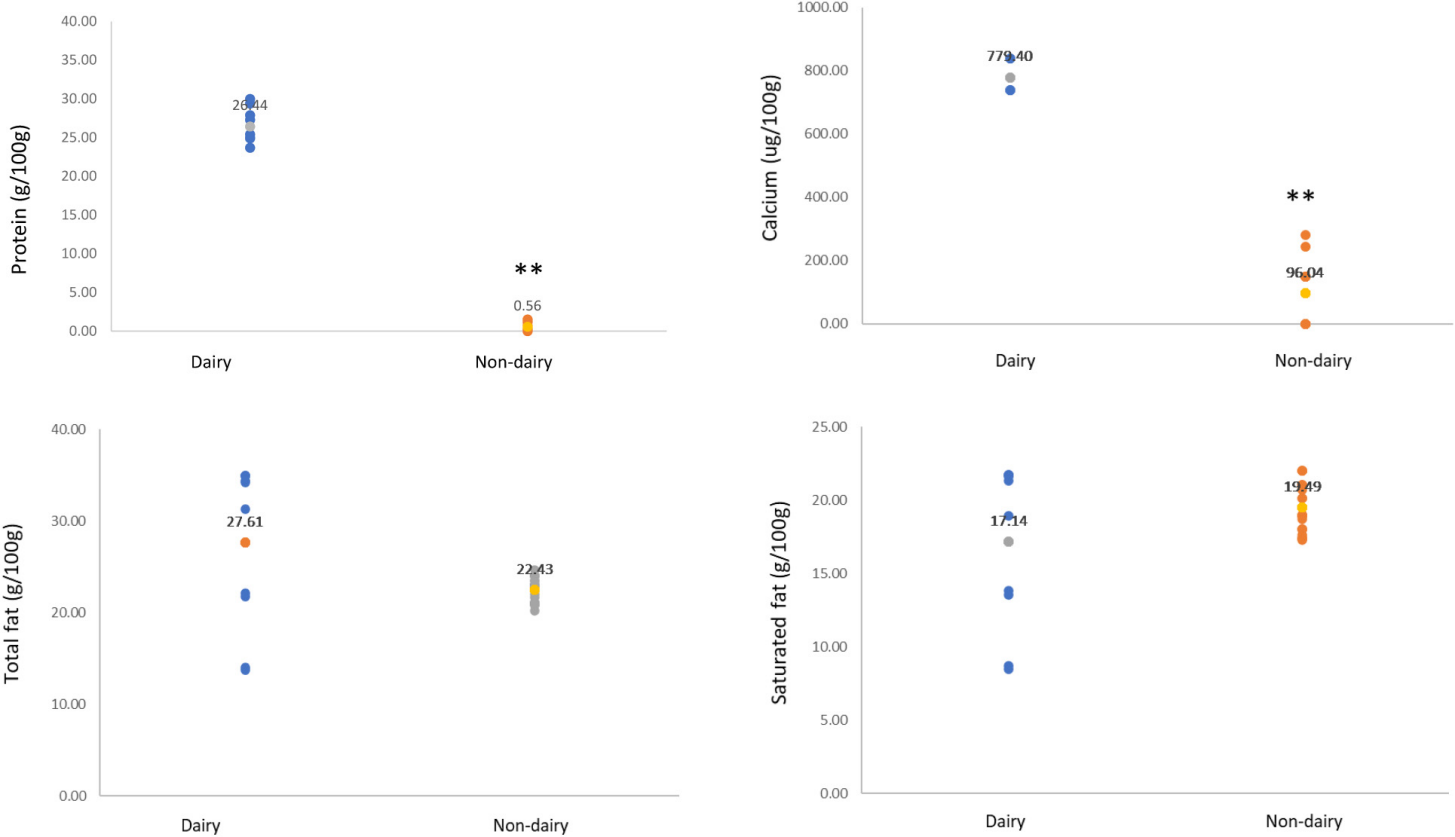
Scatter graphs of protein, calcium, total and saturated fat content of UK non-dairy cheese alternatives. Data were analyzed using Mann-Whitney comparison tests and are expressed as medians outlined with a different-colored dot and numerical values. Groups labelled with two asterisks (**) are significantly different (*p* < 0.01) from the control median (dairy cheese).

The composition of selected macro- and micronutrients present in ND cheeses and dairy, expressed as median and range per 100 g, is presented in Table 2. The distribution of key nutrients for cheese including energy, calcium, total and saturated fat, is illustrated in Figure 2, and are expressed as median values and ranges per 100 g of product. ND cheese was available at a significant higher price compared to dairy (*p* < 0.01). As >99% of samples were chilled there was no further adjustment of price for storage method, and as all sample's prices were recommended sale prices no adjustment for promotional activity was necessary. ND cheese was significantly lower in energy density and protein and significantly higher in fibre and carbohydrate content. Interestingly, ND cheese contained slightly lower levels of fat, but higher levels of saturated fat compared to dairy. ND cheese was lacking in all micronutrient content except where calcium or vitamin B12 fortification was present.

**Table 2.** Median price and selected macro- and micronutrient content per 100 g of dairy and ND cheddar in UK supermarkets.

Per 100 g serving	Cheese	Range	Non-dairy cheese	Range
Price (GBP)	0.7	0.7–1.1	1.2 **	0.7–1.7
Energy (Kcal)	403	247–416	294 **	270–305
Carbohydrate (g)	0.5	0.1–4.7	21 **	19.4–25.2
Sugars (g)	0.1	0–0.6	0.2	0–2.1
Fibre (g)	0	0–0	1.1 **	0–5.9
Salt (g)	1.8	1.8–2	1.8	1.5–2.5
Iodine (ug)	30	0–30	0 **	0–0
Potassium (mg)	75	75–110	0 **	0–0
Riboflavin (mg)	0.4	0.4–0.5	0**	0–0
Vitamin B12 (ug)	2.4	1.3–2.4	0**	0–2.5

Two (**) asterisks indicate statistically different values from the dairy control (*p* < 0.01), as analyzed by Mann-Whitney tests. GBP (Great British Pounds).

## Discussion

### Dairy milk versus ND alternatives

Table 3 presents the changes in macro- and micronutrients intake associated with replacing cow's milk with plant-based alternatives in the context of the overall diet, assuming the UK recommended intakes for cow's milk are met (3 portions daily of 200 mL). Data indicates that cow's milk is a significant source of protein, calcium, iodine, riboflavin, and vitamin B12, providing >48% of the daily recommended intake. From the ND alternatives, the macronutrient profile of oat milk is the closest to dairy, except for protein content (Table 3).

**Table 3.** Nutrient reference values for a 200 mL serving (one serving) of dairy and ND milks. Values in brackets represent % NRV values based on current UK guidelines of three dairy servings for an adult female.

Per 200 mL serving	NRV	Dairy	Almond	Soya	Oat	Coconut
Energy (kcal)	2000	98 [14.7]	42 [6.3]	66 [9.9]	91 [13.8]	40 [6]
Fat (g)	78	3.4 [13.2]	2.9 [11.1]	3.6 [13.8]	3 [11.7]	2 [7.8]
Saturated Fat (g)	24	2 [24.9]	0.1 [1.2]	0.6 [7.5]	0.4 [4.8]	1.8 [22.5]
Carbohydrate (g)	267	9.6 [10.8]	2.4 [2.7]	1.9 [2.1]	13.2 [14.7]	4.4 [5.1]
Sugars (g)	90	9.6 [31.8]	1 [3.3]	1[3.3]	8 [26.4]	3.8 [12.6]
Fibre (g)	30	0 [0]	1 [9.9]	1 [9.9]	1.5 [15]	0.2 [1.8]
Protein (g)	45	7.2 [48]	1.2 [7.8]	6.6 [43.8]	1.4 [9.3]	0.6 [3.9]
Salt (g)	6	0.2 [9.9]	0.3 [15]	0.2 [9.9]	0.2 [9.9]	0.2 [9.9]
Calcium (mg)	700	248 [106.2]	240 [102.9]	240 [102.9]	240 [102.9]	240 [102.9]
Iodine (ug)	140	64 [137.1]	0	0	0	0
Potassium (mg)	3500	324 [27.9]	30 [2.7]	86 [7.5]	74 [6.3]	28 [2.4]
Riboflavin (mg)	1.1	0.5 [136.8]	0.4 [109.2]	0.4 [109.2]	0.4 [109.2]	0
Vitamin B12 (ug)	1.5	1.8 [360]	0.8 [159.9]	0.8 [159.9]	0.8 [159.9]	0.8 [159.9]

Data in NRV column is extracted from Public Health England (2016a, 2016b).

Protein is an essential nutrient for bone health and maintaining skeletal muscle mass. Despite common belief, on average protein intakes have been documented to be lower in those following plant-based diets (Bradbury et al., 2017; Davey et al., 2003). The only ND milk with protein levels comparable to dairy was soya, providing 43.8% of the daily recommended intake (Table 3). When looking at protein quality between dairy and soya, the protein digestibility corrected amino acid score (PDCAS) suggests soya and milk protein show similar levels of 91 and 121 respectively (Schaafsma, 2000). Recent protein quality tests such as the digestible indispensable amino acid score (DIAAS) have however challenged the application of PCDAS in humans and suggest it may overestimate the quality of plant-based proteins (Mathai et al., 2017).

Calcium is an essential macro-mineral for bones and teeth, while also being an important signalling molecule in contractile tissue (Ross et al., 2011). Dairy products represent the major source of calcium in the UK, and the results of this study suggest highly significant differences between dairy and ND products (*p* < 0.01) (Table 1). All ND alternatives are calcium-fortified. However, not all calcium sources may equal bioavailability, with calcium carbonate being the primary source of fortification in the ND drinks in this research. Some evidence suggests poorer absorption rates compared to dairy (Heaney et al., 2000, 2005). Heaney et al. found calcium carbonate fortified soy milk was only absorbed at 75% of the efficiency of dairy (Heaney et al., 2000). Dairy contains lactose and casein phosphopeptides which have both been shown to increase the bioavailability of calcium, while ND milks contain compounds such as phytic acid which may further reduce calcium bioavailability (Chalupa-Krebzdak et al., 2018; Gupta et al., 2013; Zhang et al., 2020). Research suggests that individuals following exclusively plant-based diets are at higher risks of bone fractures (Hansen et al., 2018; Tong et al., 2020). Average calcium intake in vegans from a Danish population was 788 mg, close to the UK NRV, yet the group still had higher biomarkers of bone turnover factors (Hansen et al., 2018). This could be since many plant-based sources rich in calcium like green vegetables contain high levels of oxalates which significantly reduce bioavailability of dietary calcium (Melse-Boonstra, 2020).

Iodine is essential for normal brain development in children, normal thyroid function, and normal cognitive functioning (EFSA, 2010). Iodine is of particular importance during early gestation due to increases in maternal thyroid hormone levels, and deficiencies can lead to mental deformations such as cretinism and compromised brain and motor development (Lee and Pearce, 2015). Several studies conducted in the UK in pregnant women have found iodine levels to be deficient, as classified by the World Health Organization criteria (Bath et al., 2014, 2015; Knight et al., 2016). 38% of girls aged 11–18 are consuming below the lower reference nutrient intake for potassium, and 27% for iodine (Public Health England, 2016a, 2016b). The main consumers of non-dairy milks in the UK are 16–24-year-olds making up a third of the market demand, with women also being more likely to follow a plant-based diet. ND alternatives are low in iodine compared to dairy. The data in this research showed none of the retailer ND milks are currently fortified with iodine, which could mean many consumers are at risk of iodine deficiency (Table 1). Existing research comparing ND milk drinker's iodine status versus cow's milk has already found insufficient levels in ND milk drinkers, and sufficient in dairy drinkers (Dineva et al., 2020).

Milk is an important source of vitamin B12, a key nutrient for normal erythropoiesis, myelination of axons, nucleic acid synthesis and cardiovascular health (Reynolds, 2006). The EPIC Oxford study, a large prospective cohort study found that vegans had the lowest serum B12 levels compared to vegetarians and omnivores (Gilsing et al., 2010). The results of this study reported highly statistically significant differences in B12 content in milk groups, with ND alternatives having lower levels (Table 1). B12 inadequacy has also recently been explored as a factor in increased bone fracture risk, due to B12's actions on insulin-like growth factors, which when levels are low can reduce bone mineral density (Pawlak, 2021). Based on a 200 mL serving, dairy milk provides 120% NRV for vitamin B12 while all ND milks provided 53% from cyanocobalamin fortification. Due to the mean caloric value being 39% lower across ND samples, consumers would have to drink 450 mL to receive the equivalent to dairy amount of B12, which is almost half of the standard one-liter container. The resulting price difference would be 18p for dairy and 38p for ND to reach the same B12 content.

### Dairy cheese versus ND alternatives

Table 4 presents the changes in macro- and micronutrients intake associated with replacing dairy cheddar cheese with ND alternatives in the context of the overall diet, assuming the UK recommended intakes for dairy cheese are met (3 portions daily of 30 g). Similarly, with the data presented for milk, dairy cheddar cheese is a significant source of protein, calcium, and vitamin B12, providing >50.4% of the daily recommended intake (Table 4). Non-surprisingly, dairy cheese is also a source of fat and particularly saturated fat. On the other hand, ND alternatives were less energy dense, with lower fat levels but very low in protein content and lacking the majority of micronutrients, due to the absence of fortification.

**Table 4.** Nutrient reference values for a 30 g serving (one serving) of dairy and ND cheddar cheese. Values in brackets represent % NRV values based on current UK guidelines of three dairy servings for an adult female.

Per 30 g serving	NRV	Dairy	Non-dairy cheese
Energy (kcal)	2000	121 [18]	88 [13.2]
Fat (g)	78	9.8 [37.8]	6.9 [26.7]
Saturated Fat (g)	24	5.1 [63.8]	5.7 [71.3]
Carbohydrate (g)	267	0.2 [0.3]	6.3 [7.2]
Sugars (g)	90	>0.1 [>0.3]	0.1 [0.3]
Fibre (g)	30	0	0.3 [3]
Protein (g)	45	7.6 [50.4]	0.2 [1.2]
Salt (g)	6	0.5 [24.9]	0.5 [24.9]
Calcium (mg)	700	222 [95.1]	45 [19.2]
Iodine (ug)	140	9 [19.2]	0
Potassium (mg)	3500	22.5 [1.8]	0
Riboflavin (mg)	1.1	0.1 [27]	0
Vitamin B12 (ug)	1.5	0.7 [139.8]	0

Data in NRV column is extracted from Public Health England (2016a, 2016b).

A notable feature of plant-based diets is, on average a lower consumption of saturated fatty acids (SFA) compared to omnivores, which could be an important factor contributing to an observed lower risk of CVD outcomes and mortality (Clarys et al., 2014). SFA have been consistently demonstrated to raise serum low density lipoprotein (LDL), which are causally associated with CVD mortality (Borén et al., 2020; Clifton and Keogh, 2017; Kim et al., 2019). The only study has directly compared ND to dairy in a CVD risk context and found that dairy cheese resulted in lower incremental area under the curve for C-reactive protein compared to dairy-free cheese (Demmer et al., 2016). Although high in SFA, many large studies have shown that dairy consumption, including full-fat products do not have the adverse effect on blood lipids and CVD risk that have been shown for other SFA rich foods such as coconut oil (Astrup et al., 2019; Briggs et al., 2017; Fontecha et al., 2019). Although no statistically significant differences were observed between SFA or total fat content between ND cheeses and dairy (Figure 2), the base ingredient for all ND cheeses was coconut oil. The latter is shown to significantly raise serum LDL concentrations due to its high SFA content (Eyres et al., 2016; Neelakantan et al., 2020). A 30 g serving of both ND cheese and dairy cheese provided similar SFA levels; however, to obtain equivalent levels of calcium (222 mg) to a 30 g serving of dairy, 147 g of ND cheese would need to be consumed which would result in 116% of the SFA recommended intake (Table 4).

48% of ND cheese samples were not fortified with calcium, including none of the market leader (Violife) products (Figure 2). ND cheeses were not fortified with potassium, iodine, riboflavin, or vitamin B12, of which a 30 g serving of dairy cheese provides 1.8%, 19.2%, 27% and 139.2% respectively of the NRV (Table 4). Both calcium and vitamin B12 are key nutrients of concern that may be limiting for those following plant- based diets (Davey et al., 2003). With the perception of plant-based diets being a healthier choice, consumers may consider ND cheeses a better choice under the pretense of being healthier, but their healthfulness within a plant-based dietary pattern has yet to be elucidated.

### Strengths and limitations of the study

Prior literature in this topic has not adequately researched the nutritional profile of ND milk products or ND cheese products, so this work aims to offer a more complete nutritional view of the ND product market in the UK.

This was not a lab-based project so analytical micronutrient values could not be calculated when missing and relied on predictive calculations. Not all different types of ND cheeses were included, which may have led to a smaller sample size for ND cheeses; however, all ND cheddar cheeses available in the major UK grocers as of May 2021 were included in the analysis and a large sample was collected. Our study did include >93% of the UK market of ND cheeses and a >80% of ND milks, including two of the largest brands and the 6 major UK grocers OL, providing a strong representative sample.

## Conclusion

Many questions remain about the intrinsic benefits of plant-based versus healthy omnivorous diet patterns. The main purpose of this research was to establish and compare the nutritional content and price of ND milk and cheese alternatives, which are growing rapidly in consumption in the UK market. Dairy products provided richer sources of key micronutrients than ND, particularly for iodine in milk and calcium and B12 in cheese. Dairy products were also on average higher in protein and calories with no significant differences in total fat content. ND milk products are similarly priced to dairy, but ND cheese is significantly more expensive than dairy cheese.

Whole food plant-based diets have a large evidence base displaying beneficial health effects. As more people look towards plant-based eating, the demand for alternative products will likely continue to grow. This work has demonstrated that compared to dairy, ND products fall short in several key nutrients. Fortification of such products is important to ensure nutritional recommendations per serving can be met, while cost and bioavailability need to be maintained at comparable levels. Finally, clear and consistent labelling should be implemented to help consumers make informed choices for healthy and balanced diets.
